# Screening for older inpatients at risk for long length of stay: which clinical tool to use?

**DOI:** 10.1186/s12877-019-1165-4

**Published:** 2019-06-06

**Authors:** Olivier Beauchet, Shek Fung, Cyrille P. Launay, Liam Anders Cooper-Brown, Jonathan Afilalo, Paul Herbert, Marc Afilalo, Julia Chabot

**Affiliations:** 1 Department of Medicine, Division of Geriatric Medicine, Sir Mortimer B. Davis - Jewish General Hospital and Lady Davis Institute for Medical Research, McGill University, 3755 chemin de la Côte-Sainte-Catherine, Montréal, QC H3T 1E2 Canada; 2 Dr. Joseph Kaufmann Chair in Geriatric Medicine, Faculty of Medicine, McGill University, Montreal, Quebec Canada; 3 Centre of Excellence on longevity of McGill integrated University Health Network, Montreal, Quebec Canada; 4 Lee Kong Chian School of Medicine, Nanyang Technological University, Singapore, Singapore; 5 Department of Medicine, Division of Geriatric Medicine, St. Mary’s Hospital Center, McGill University, Montreal, Quebec Canada; 6 Geriatric Medicine and Geriatric Rehabilitation ServiceDepartment of Medicine, Lausanne University Hospital, Lausanne, Switzerland; 7 Division of Cardiology and Centre for Clinical Epidemiology, Jewish General Hospital and McGill University, Montreal, Quebec Canada; 8 Department of medicine, Montreal university Hospital and University of Montreal, Montreal, Quebec Canada; 9 Emergency Department, Jewish General Hospital, McGill University, Montreal, QC Canada

**Keywords:** Older inpatients, Epidemiology, Screening, Frailty

## Abstract

**Background:**

Screening for inpatients at risk for long length of stay (LOS) is the first step of an effective hospital care plan for older inpatients. This study aims, in older adults admitted to a geriatric acute care ward, to examine and compare the 6-item brief geriatric assessment (BGA) and the “*Programme de Recherche sur l’Intégration des Services pour le Maintien de l’Autonomie*” (PRISMA-7) risk levels with long LOS, and to establish their performance criteria (i.e., sensitivity, specificity, positive predictive value, negative predictive value, likelihood ratios) for LOS.

**Methods:**

Based on an observational, retrospective, cohort design, 166 inpatients aged ≥75 admitted to a geriatric acute care ward of a McGill University-affiliated hospital (Montreal, Quebec, Canada) were recruited. The risk levels of the 6-item BGA (low, moderate and high) and the PRISMA-7 (low versus high) were calculated from a baseline assessment. The LOS was subsequently calculated in number of days.

**Results:**

Only the 6-item BGA high risk level was associated with a long LOS (Odds ratio = 1.1 with *P* = 0.028 and Hazard ratio = 2.1 with *P* = 0.004). Kaplan-Meier distributions showed that there was no significant difference in the delay of hospital discharge between the low and high-risk level reported by the PRISMA-7 (*P* = 0.381), whereas the 6-item BGA three risk levels differed significantly (*P* = 0.008), with individuals at high risk levels being discharged later when compared to those with low (*P* = 0.001) and moderate (*P* = 0.019) risk levels. Both tools’ performance criteria were poor (i.e., < 0.70), except for PRISMA-7’s sensitivity which was 100%.

**Conclusion:**

The 6-item BGA risk levels were associated with LOS, low risk-level being associated with short LOS and high-risk level with long LOS, but no association was reported with the PRISMA-7 risk levels. Both tools had poor performance criteria for long LOS, suggesting that they cannot be used as prognostic tools with current scientific knowledge.

## Background

Older (over the age of 65) inpatients are major users of hospital-based acute care services and are a growing group among patients admitted to acute care wards [1–4]. Most of them are admitted to acute care after an Emergency Department (ED) visit [1, 2]. Instead of one straightforward acute disease, older patients present a complex combination of multiple comorbid conditions, which greatly influences their care plans [3, 4]. For instance, although older inpatients undergo more diagnostic tests and procedures than younger inpatients, their diagnoses tend to be less accurate, slowing down the care plan’s design [3–6]. Comorbid conditions may also decompensate in cascade and lead to disabilities, increasing an inpatient’s length of stay (LOS) [5, 6]. Consequently, the LOS of older inpatients is longer than younger inpatients’, contributing - against the backdrop of a reduction in the number of hospital beds - to hospital overcrowding [3]. Reduction of LOS is, therefore, one of the main challenges that hospitals face today.

Developing rapid screening methods to estimate the risk of long LOS in older inpatients is relevant, as these can lead to simple and protective subsequent interventions [3, 5, 7, 8]. Screening older inpatients with a high risk for long LOS upon ED visits or admissions to acute care wards is, thus, the first step of a hospital care plan that is effective in this population [3, 7, 8]. Several clinical tools have been proposed, but most of them screen for frail conditions or frailty-related issues and adverse events after hospital discharge [9]. Few tools have been designed to screen for the risk of adverse events upon hospital admission and in acute care wards [9]. The only prognostic tool for long LOS is the 6-item brief geriatric assessment (BGA) [7–20]. The 6-item BGA provides a three risk levels for LOS (i.e., low, moderate and high) [7, 8, 19]. The 6-item BGA meets most of the requirements (i.e., fast, easy to use, standardized and based on clinical information collected early in the care process) for being an effective screen of short-term hospital adverse outcomes like long LOS [16, 19, 21]. In Quebec, the “*Programme de Recherche sur l’Intégration des Services pour le Maintien de l’Autonomie*” (PRISMA-7) is the Ministry of Health and Social Services’ reference tool for use in EDs and in acute care wards [14, 15]. This tool, which stratifies older patients into two disability risk levels (i.e., low versus high), has never had its predictive value of long LOS risk validated. Thus, examining the association between the risk levels reported by the PRISMA-7 and long LOS and comparing them with how the 6-item BGA risk levels are associated with long LOS could be helpful to choose the correct prognosis tool to use. We hypothesized that the 6-item BGA would be better than the PRISMA-7 at predicting long LOS, as it has been designed solely to predict this adverse hospital outcome in acute care wards. This study aims to examine and compare the respective associations of risk levels generated by the 6-item BGA and by the PRISMA-7 with LOS and evaluate both tools’ performance criteria (i.e., sensitivity, specificity, positive predictive value [PPV], negative predictive value [NPV], likelihood ratios [LR]) for LOS in older adults admitted to a geriatric acute care ward.

## Methods

### Participants

Older inpatients consecutively admitted to a geriatric acute care ward in a university-affiliated hospital were enrolled in this observational retrospective cohort study. The recruited participants were a subgroup of participants enrolled in a previous study [22]. The aim of this first study was to examine whether the 6-item BGA levels of risk for long LOS successfully predicted long LOS in Quebecois geriatric inpatients admitted to a geriatric acute care ward. The period of recruitment was October 2014 to May 2016. The inclusion criteria were: 1) having received both 6-item BGA and PRISMA-7 scores, 2) admission to a geriatric acute care ward after an ED visit and 3) LOS ≤ 2 months. This threshold of 2 months was established because geriatric acute care wards are designed to take care of acute inpatients and there is no reference average LOS in Quebec’s wards. Additionally, inpatients with a LOS above 2 months are administratively reclassified as inpatients in stable medical condition who are waiting for placement in either residences or long-term care facilities. A total of 166 (33.3%) out of the initial cohort’s 499 participants were identified based on selection criteria and selected for this study. Assuming a power of 90% and a two-sided alpha of 0.5, the power analysis showed that 150 individuals needed to be recruited to achieve the objective of this second study.

### Assessment

The answers to the 6-item BGA and PRISMA-7 items were collected from patients’ medical charts. Both tools’ items were completed by nurses upon participant admission to the acute care ward during a short clinical assessment that included a face-to-face interview with patients. Regardless of the tool used, items were answered using a closed-ended format (i.e., yes or no). Based on the answers, points were assigned to each item and a score was calculated.

The items of the BGA are as follows: age ≥ 85; male; polypharmacy, defined as ≥5 different medications taken daily; presence of home support provided by a health care or social professional, and/or family and/or friend; history of falls in the past 6 months; and temporal disorientation, defined as an inability to give the current month and/or year. 5 points were assigned to history of falls and temporal disorientation and 1 point was assigned to the four other items, as per the results of our previous studies [22]. The score ranged from 0 (lowest risk) to 14 (highest risk) and enabled stratification of the risk for long LOS into three levels: low (score 0 to 3), moderate (score 4 to 5) and high (score ≥ 6). The other tool, PRISMA-7, is composed of 7 questions, which are as follows: 1) Are you more than 85 years old?; 2) Are you male?; 3) In general, do you have any health problems that require you to limit your activities?; 4) Do you need someone to help you on a regular basis?; 5) In general, do you have any health problems that require you to stay at home?; 6) In case of need, can you count on someone close to you?; and 7) Do you regularly use a stick, walker, or wheelchair to move about?. The scoring method used by the PRISMA-7 is to assign 1 point to the answer *yes* and no points to the answer *no*. It stratifies inpatients into two disability risk groups: low (score 0 to 2) and high (score 3 to 7) risk. Physicians and patients were kept blind to the scores and associated risk levels derived from the 6-item BGA and the PRISMA-7. Reasons for admission were classified into four main categories, which were mobility disorders (i.e., gait and/or balance disorders and/or fall), neuropsychiatric disorders (i.e., delirium, dementia or behavioral disorders), organ failure (i.e., acute organ decompensation) and social issues (i.e., absence of acute diseases accompanied by an inability to cope with increased formal and/or informal home and social support).

### Outcomes

LOS was calculated from the hospital’s administrative registry. It was defined as the delay (in days) between the first day of admission to the geriatric acute care ward to the day of discharge from the hospital. A long LOS was defined as one that is in the highest tertile of LOSs (i.e., > 13 days).

### Standard protocol approvals, registrations, and participant consent

Verbal informed consent was obtained from all participants by a systematic and standardized process used in the geriatric acute care ward where the study was performed. Participants or their legal guardians, when appropriate, were informed that their medical information may be used for research purposes. If they disagreed, they informed the physician taking care of them and a note was recorded in their chart. The ethics committee approved this process. No refusal was recorded for this study. The ethics committee of St. Mary’s Hospital (Montreal, Quebec, Canada) approved this study.

### Statistical analysis

Means and standard deviations (SD) or frequencies were used to summarize the participants’ baseline characteristics. Between-group comparisons were performed using an analysis of variance (ANOVA) with Bonferroni corrections for multiple comparisons, unpaired t-tests or Chi-square tests, as appropriate. Multiple linear, logistic and Cox regression models were used to study the association of LOS (dependent variable) with the risk levels (independent variable) derived from either the 6-item BGA (i.e., low, moderate, high) or the PRISMA-7 (i.e., low versus high). The low risk level was used as a reference level and an adjustment for reasons for admission was performed in all models. The elapsed time between admission to and discharge from the geriatric acute care ward was also examined for the different risk groups (low versus high for PRISMA-7 and low, moderate or high for 6-item BGA) by survival Kaplan-Meier curves and log-rank tests. The considered performance criteria were sensitivity, specificity, PPV, NPV and LR. *P*-values < 0.05 were considered statistically significant. All statistics were performed using SPSS (version 23.0; SPSS, Inc., Chicago, IL).

## Results

Table 1 shows participants’ baseline characteristics and comparisons between the groups established via the PRISMA-7 and the 6-item BGA risk levels. The PRISMA-7’s high-risk group had higher frequencies of home support (*P* ≤ 0.001) and polypharmacy (*P* = 0.002) than its low-risk group. The 6-item BGA’s moderate-risk group was older (*P* = 0.004) and had a higher prevalence of temporal disorientation (*P* = 0.016) and history of falls (*P* ≤ 0.001) than its low-risk group. The prevalence of temporal disorientation (*P* ≤ 0.001) and histories of falls (*P* ≤ 0.001) as well the mean LOS (*P* = 0.001) were higher in the high-risk group when compared to the low-risk group. In addition, there were more males (*P* = 0.002), more temporal disorientation (*P* ≤ 0.001) and higher mean LOS (*P* = 0.013) in the high-risk group compared to moderate-risk group. There was no significant difference in the other evaluated characteristics.

**Table 1:** Baseline characteristics and comparisons between groups of participants categorized with the risk-level stratification of the PRISMA-7 (i.e. low versus high) and the 6-item BGA (i.e. low, moderate and high) (*n* = 166)

Baseline Characteristics	Total population (*n* = 166)	PRISMA-7 score		*P*-Value^†^	6-item BGA risk levels			*P*-Value^‡^			
		Normal (*n* = 22)	Abnormal* (*n* = 144)		Low (*n* = 58)	Moderate (*n* = 85)	High (*n* = 23)	Overall	Low versus moderate risk	Low versus high risk	Moderate versus high risk
Age ≥ 85 years, n (%)	105 (63.3)	11 (50.0)	94 (65.3)	0.166	45 (77.6)	46 (54.1)	14 (60.9)	**0.016**	**0.004**	0.127	0.563
Male, n (%)	41 (24.7)	2 (9.1)	39 (27.1)	0.068	16 (27.6)	14 (16.5)	11 (47.8)	**0.007**	0.109	0.081	**0.002**
Home support^||^, (%)	133 (80.1)	10 (45.5)	123 (85.4)	≤**0.001**	47 (81.0)	64 (75.3)	22 (95.7)	0.093	–	–	–
Polypharmacy^¶^, n (%)	137 (82.5)	13 (59.1)	124 (86.1)	**0.002**	46 (79.3)	72 (84.7)	19 (82.6)	0.706	–	–	–
Temporal disorientation^§^, n (%)	26 (15.7)	3 (13.6)	23 (16.0)	0.779	0 (0.0)	8 (9.4)	18 (78.3)	≤**0.001**	**0.016**	≤**0.001**	≤**0.001**
History of falls^#^, n (%)	100 (60.2)	11 (50.0)	89 (61.8)	0.292	0 (0.0)	77 (90.6)	23 (100.0)	≤**0.001**	≤**0.001**	≤**0.001**	0.126
Reason for admission, n (%)											
Mobility disorders**	107 (64.5)	13 (59.1)	94 (65.3)	0.298	37 (63.8)	57 (67.1)	13 (56.5)	0.371	–	–	–
Neuropsychiatric disorders^††^	25 (15.1)	4 (18.2)	21 (14.6)		8 (13.8)	14 (16.5)	3 (13.0)		–	–	–
Organ failure||||	15 (9.0)	4 (18.2)	11 (7.6)		3 (5.2)	8 (9.4)	4 (17.4)		–	–	–
Social issue^¶¶^	19 (11.4)	1 (4.5)	18 (12.5)		10 (17.2)	6 (7.1)	3 (13.0)		–	–	–
Length of stay^§§^ (days)											
Mean ± SD	14.1 ± 13.8	11.2 ± 12.6	14.5 ± 14.0	0.298	11.1 ± 9.9	13.8 ± 14.0	22.9 ± 18.2	**0.002**	0.707	**0.001**	**0.013**
> 13^##^, n (%)	56 (33.7)	4 (18.2)	52 (36.1)	0.98	15 (25.9)	29 (34.1)	12 (52.2)	0.078	–	–	–

PRISMA-7: the seven questions “Program of Research on the Integration of Services for the Maintenance of Autonomy”

*BGA* Brief geriatric assessment

*SD* standard deviation

*Score ≥ 3/7

^†^Comparison based on unpaired *t*-test or Chi-square test, as appropriate

^‡^Comparison based on ANVOA with Bonferroni correction for multiple comparisons or Chi-square test, as appropriate

^||^Use of formal (i.e., health and/or social professional) and/or informal (i.e., family and/or friends) home services

^¶^Number of therapeutic classes taken daily ≥5

^§^Inability to give the month and/or year

^#^in the previous 12-month period

**Defined as gait and/or balance disorders and/or fall defined as unintentionally coming to rest on the ground, floor, or other lower level

^††^Defined as delirium, dementia or behavioral disorders

^||||^Defined as an acute organ decompensation

^¶¶^Defined as the absence of acute disease symptoms combined with an acute increase of the use of formal and/or informal home and social services, leading to an inability to stay at their place of residence for life

^§§^Based on the hospital’s administrative registry. Corresponds to the delay in days between the first day of admission to geriatric ward and the last day of hospitalization in the same geriatric ward

^##^Corresponding to the highest tertile among lengths of stay

*P*-value significant (i.e.; < 0.05) indicated in bold

**Fig. 1:**
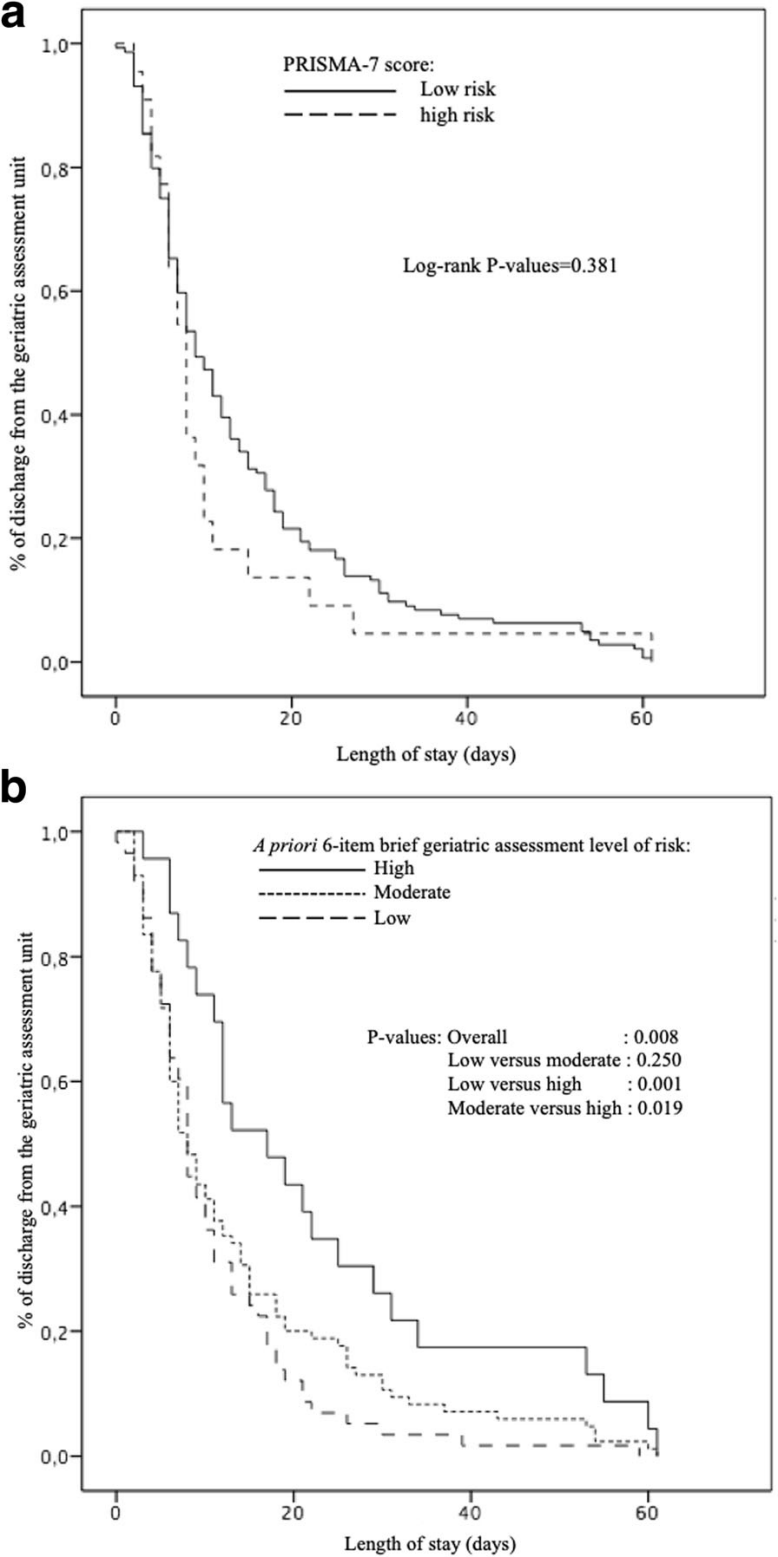
Kaplan-Meier estimates of the probability of discharge from the geriatric acute care ward based on PRISMA-7 risk levels (i.e.; low versus high) (**a**) and on the 6-item BGA risk levels (i.e.; low, moderate, high) (**b**) (*n* = 166). PRISMA-7: the seven questions “Program of Research on the Integration of Services for the Maintenance of Autonomy”. BGA: Brief geriatric assessment

Only the 6-item BGA risk levels were significantly associated with LOS in linear regressions (Table 2). The low risk level was associated with a short LOS (coefficient of regression β = − 4.9 with *P* = 0.030) and the high risk level was associated with a long LOS (coefficient of regression β = 9.9 with *P* = 0.001). Multiple logistic and Cox regressions showed that only the 6-item BGA high risk level was associated with a long LOS (Odds ratio = 1.1 with *P* = 0.028 and Hazard ratio = 2.1 with *P* = 0.004). When it comes to the PRISMA-7 risk levels, Kaplan-Meier distributions showed no significant differences in the time elapsed before hospital discharge between low- and high-risk groups (*P* = 0.381). In contrast, the three participant risk groups established with the 6-item BGA differed significantly when it comes to LOS (*P* = 0.008), with those in the high-risk group being discharged later than those in the low-risk (*P* = 0.001) and moderate-risk (*P* = 0.019) groups (Fig. 1). All performance criteria were poor (i.e., < 0.70), except the sensitivity of PRISMA-7, which was 1 (Table 3).

**Table 2:** Multiple regressions showing the association of length of stay in days (dependant variable) with the PRISMA-7 high risk level and the 6-item BGA risk levels (i.e., low, moderate and high) (independent variable, separated model for the PRISMA-7 and the 6-item brief geriatric assessment) adjusted with reasons for admission (*n* = 166)

	Linear regression*			Logistic regression^†^			Cox regression*		
	ß	[95% CI]	*P*-value	OR	[95% CI]	*P*-value	HR	[95% CI]	*P*-value
PRISMA-7 Abnormal score	3.3	[−2.9;9.6]	0.295	2.6	[0.8;7.9]	0.107	1.2	[0.8;1.9]	0.398
A priori 6-item BGA levels of risk									
Low	**−4.9**	[−9.3;-0.5]	**0.030**	Ref.			Ref.		
Moderate	−0.3	[− 4.6;4.0]	0.887	0.4	[0.7;3.2]	0.279	1.3	[0.9;1.8]	0.195
High	**9.9**	[4.0;15.9]	**0.001**	**1.1**	[1.1;8.5]	**0.028**	**2.1**	[1.3;3.4]	**0.004**

PRISMA-7: the seven questions “Program of Research on the Integration of Services for the Maintenance of Autonomy”

*BGA* Brief geriatric assessment

*ß* Coefficient of regression beta

*OR* Odds ratio

*HR* Hazard ratio

*CI* Confidence interval

*Ref* Referent condition

*Length of stay used as a continuous dependant variable

^†^Length of stay used as a discontinuous variable defined as the highest tertile of lengths of stay (i.e., > 13 days)

All models adjusted with reason for admission, including mobility disorders (i.e., gait and/or balance disorders and/or fall, defined as unintentionally coming to rest on the ground, floor, or other lower level), neuropsychiatric disorders (i.e., delirium, dementia or behavioral disorders), organ failure (i.e., acute organ decompensation) or social issue (i.e., absence of symptoms of acute diseases combined with an acute increase of the use of formal and/or informal home and social services, leading to an inability to stay at their place of residence for life)

Coefficient of regression beta, odds ratio, hazard ratio and *P*-value significant (i.e.; < 0.05) indicated in bold

**Table 3:** Performance criteria of risk levels of the 6-item brief geriatric assessment and the PRISMA-7 for long lengths of hospital stay* (*n* = 166)

Performance criteria	6-item brief geriatric assessment risk level		Abnormal PRISMA-7 score^†^
	Moderate	High	
Sensitivity	0.56	0.23	1.00
Specificity	0.08	0.14	0.03
Positive predictive value	0.34	0.52	0.36
Negative predictive value	0.67	0.69	0.82
Likelihood ratio of positive test	0.60	0.27	1.03
Likelihood ratio of negative test	0.17	0.18	–

PRISMA-7: the seven questions “Program of Research on the Integration of Services for the Maintenance of Autonomy”

*Length of stay used as a discontinuous variable defined as the highest tertile of lengths of stay (i.e., > 13 days)

^†^Score ≥ 3/7

## Discussion

The findings show that the 6-item BGA risk levels were associated with LOS, with the low-risk level associated with short LOS and the high-risk level associated with long LOS, but no significant association was reported when using the PRISMA-7 risk levels. In addition, performance criteria were poor for both tools.

The association of the 6-item BGA risk levels with LOS has previously been demonstrated [19, 20]. However, this association was reported in a French older patient cohort [16, 19, 21]. To date, only one study performed in Quebec showed an association between BGA risk levels and LOS, with high risk levels being associated with long LOS [22]. The 6-item BGA is a prognostic tool for screening short-term frailty-related adverse events. Frailty may be defined as an inability to respond appropriately to stressors like an admission to hospital and is caused by the reduction in physiological resources due to the combined actions of aging and morbidities [4, 5, 23, 24]. Thus, frail inpatients require more time to re-attain their baseline conditions, which explains their increased risk of long LOS relative to non-frail inpatients [5, 19, 20]. Given this context, the early detection of possibly frailer inpatients who are at risk for long LOS becomes a cornerstone of older inpatient care. Substantial resources are devoted to reducing the duration and severity of acute diseases in acute wards, but multi-morbidities and disabilities are more likely to be ignored [3]. This last point explains why older inpatients require more staff time and resources than younger inpatients [1–3, 5] and, thus, are more prone to short-term adverse outcomes like long LOS. This short-term adverse outcome is one of the major challenges in the demand for hospital services and calls for new care strategies that maintain an effective and efficient hospital care system. Hospital health professionals who take care of older patients are concerned with being able to identify the right patients (i.e., those most at risk of adverse outcomes) at the right time (i.e., as soon as possible) and to introduce the right intervention (i.e., appropriate to the patients’ health and functional conditions) with the goal of reducing the occurrence of short-term adverse outcomes [3]. The best way to prevent/reduce adverse outcomes in hospital is to screen those older patients at the highest risk as soon as possible (i.e., earlier in the care plan) such that they receive timely and appropriate interventions [1, 2].

The predictive tools designed for this purpose should provide a relevant stratification of risk and information for the hospital care plan early [1, 2]. The use of clinical information has been shown to be the better strategy in the development of predictive tools when compared to administrative data collection [1, 2, 6, 10–12]. As such, this approach has generated several screening tools which: 1) use the same categories of variables, including demographic, social, physical and functional parameters; 2) are based on information collected via questions asked to patients and/or their relatives, instead of recorded via physical examination; 3) provide a score based on an accumulation of points wherein all items have the same value; 4) and provide two levels of risk: low versus high [7–20]. Among these screening tools, most like ISAR and TRST have been developed for EDs [8–13]. In Quebec, the PRISMA-7 is the reference tool provided by the Ministry of Health and Social Services of Quebec [14, 15]. However, this tool has only been validated to screen for disabilities and only in older community dwellers whose medical conditions are stable [14, 15]. The PRISMA-7 is not easy to implement in hospitals because its questions do not always lead to objective answers, particularly when it comes to acute conditions. Indeed, it requires collecting information on the patient’s history by asking questions to the patients themselves or to their relatives. This can sometimes lead to subjective/inaccurate answers that do not reflect the patient’s real status. In addition, due to the increased prevalence of cognitive impairment among older users of acute care wards, obtaining reliable information is all the more difficult [6–10]. Another weakness of the PRISMA-7 is that it only predicts medium-term age-related adverse outcomes like disability, whereas in-hospital professionals need relevant information about short-term adverse outcomes [3]. No association between the PRISMA-7 high risk level and LOS has been reported in our results, suggesting that the PRISMA-7 is not an appropriate tool to screen age-related short-term adverse events in older inpatients admitted to acute care wards.

Both the 6-item BGA and the PRISMA-7 have a limited number of items that are not specific to older inpatients. In addition, we showed that their performance criteria were poor, suggesting that they cannot be used as prognosis tool for long LOS with current scientific knowledge. Taking into consideration the reasons for admission or increasing the number of items could improve the predictive value of these clinical tools. We examined this hypothesis for the 6-item BGA in a previous study [20]. We suggest that increasing the number of items found on the BGA from 6 to 10 could improve its accuracy and performance criteria for predicting long LOS. We demonstrated that the performance criteria of the 10-item BGA for the prediction of long LOS were higher than the 6-item BGA [20]. We also highlighted that chronic conditions were the main contributors to a good predictive value. However, a tool’s usability is important to take into consideration. Increasing the number of items tends to decrease tools’ usability. The choice of including only 6 items in the BGA has been made in order to take into account “real-life” conditions of practice. In fact, it has been shown that screening tools must be completable in less than 5 min to be considered usable in daily practice [25].

There are limitations to our study. First, the recruitment of participants was performed in a single centre and the number of participants recruited was a small sample. Thus, the studied cohort is not representative of inpatients admitted to acute care wards at the population level, which limits the results’ external validity. Second, the retrospective collection of data from patient medical charts limited what information was collected. It could be suggested that other, non-collected variables may influence the relationship between the 6-item BGA and the PRISMA-7’s risk levels and LOS. Third, a potential recall bias may affect the reported history of falls. Indeed, falls are more frequently underreported in older adults than in younger adults, because memory impairment can lead this population to forget the occurrence of falls [26].

## Conclusions

The 6-item BGA risk levels was associated with LOS in older inpatients admitted to acute care wards, with older adults classified as high-risk having a greater risk of long LOS when compared to the two other risk levels. In contrast, the PRISMA-7 failed to identify those older inpatients who underwent long LOS. However, both tools had poor performance criteria for long LOS, suggesting that they cannot be used as prognostic tools with current scientific knowledge.

Further research is needed to confirm this first result.

## Data Availability

The datasets used and analyzed in the current study will be made available by the corresponding author upon reasonable request.

## Abbreviations

ANOVA: Analysis of variance
BGA: Brief geriatric assessment
ED: Emergency Department
LOS: Length of stay
PRISMA-7: “*Programme de Recherche sur l’Intégration des Services pour le Maintien de l’Autonomie*”
SD: Standard deviation
